# Can you learn not to respond to irrelevant motion while making fast arm movements?

**DOI:** 10.1371/journal.pone.0332171

**Published:** 2025-09-19

**Authors:** Eli Brenner, Lara M. de Jonge, Niels J. J. Tiems, Glenn Rosenquist, Thijs Wiggers, Jeroen B. J. Smeets, Emily M. Crowe

**Affiliations:** 1 Department of Human Movement Sciences, Vrije Universiteit Amsterdam, Amsterdam, The Netherlands; 2 School of Psychology, University of Nottingham, University Park, United Kingdom; University of Muenster, GERMANY

## Abstract

If the target of a goal-directed arm movement is suddenly displaced, the movement is quickly adjusted in accordance with the target’s new position. Such quick adjustments are even observed if the target is moving, so the arm is moving towards a future target position. If irrelevant items near that future position suddenly start moving, the arm produces a futile response in the direction of the irrelevant items’ motion. We wondered whether people could learn not to respond to such irrelevant motion. To find out, we had participants intercept targets that moved across a background consisting of hundreds of small dots (irrelevant items). At a fixed time before the target reached an interception zone, the background dots started moving. The response to this irrelevant motion declined across the first few hundred trials, but it never fully disappeared. Including trials in which the target jumped established that participants learnt to reduce their response to the irrelevant motion, rather than to reduce their response to any new information. We conclude that participants can reduce the extent to which they respond to irrelevant motion, but that they cannot suppress such responses altogether.

## Introduction

When reaching out for an object, one’s movement is constantly guided by the latest information about the object and about the moving arm [1]. Such guidance is often studied by suddenly displacing the target of a goal-directed arm movement [2,3] or a cursor representing the fingertip [4–8]. The response is fast and automatic. People do not need to detect the displacement to respond to the new position [9,10] and they can respond to dozens of new positions during a single movement [11,12]. Moreover, they cannot avoid responding when instructed not to [13] or when doing so is detrimental [14].

If the target that one is trying to intercept is moving, one’s movement is directed towards a position that the target will reach in the future. If irrelevant items near that future target position suddenly start moving in a certain direction, the arm follows their movement in that direction [15]. This is even so if the items are part of a large background that is clearly irrelevant [16,17]. Is this response to irrelevant motion part of an automatic system that is essential for guiding the arm to the correct position [18]? If it is useful, but not essential, people might learn to respond less to such sudden irrelevant motion when responding is less advantageous.

Many studies investigating how ongoing movements are affected by the sudden motion of irrelevant items were designed in a way that could be expected to discourage participants from learning not to respond to such motion. For instance, several studies randomly interleaved trials with and without irrelevant motion [19–21]. On trials where there is no irrelevant motion, responding to retinal motion near the planned movement endpoint may help update that endpoint’s position when one’s eyes and head move, which could help guide the hand to the planned endpoint [18]. So, including trials without irrelevant motion might discourage people from suppressing responses to retinal motion in the background, because it might not always be advantageous to do so. Another choice that might discourage participants from learning not to respond to irrelevant motion is removing the target before the irrelevant motion starts [21–23], so that the movement is directed toward a remembered position rather than a visible one. Not seeing the target presumably makes it more difficult to judge one’s error when the hand follows the irrelevant motion. Both these choices might prevent participants from learning not to respond to irrelevant motion. But irrelevant motion also influences arm movements when it is present on all trials [16,17] and when the arm movement is directed towards targets that remain visible at a fixed position [2,20].

Thus, although many researchers appear to have considered the possibility that people can learn to respond less vigorously to the sudden onset of irrelevant motion, such learning has never explicitly been studied. This paper set out to determine to what extent people can learn not to respond to sudden onset of irrelevant motion if such motion is presented at the same moment on hundreds of successive trials. Our first question was whether people can learn to suppress the response to irrelevant motion. If they do learn to suppress the response, a second interesting question is whether they learn to specifically suppress responses to irrelevant motion, or whether they can only learn to suppress responding to all visual information that is presented during the movement. We therefore also examined responses to the target unexpectedly jumping to a new position. In our first experiment, we compared responses to target jumps before and after exposure to irrelevant motion, ensuring that the sequence of trials with irrelevant motion was not interrupted. In two further experiments we examined how interleaving trials with irrelevant motion with trials with relevant target jumps influences learning not to respond vigorously to the onset of irrelevant motion.

## Methods

**Fig 1:**
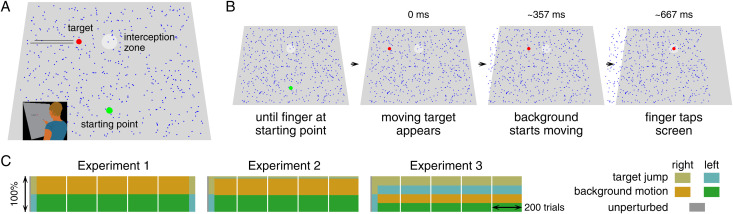
The task and conditions. (A) Participants stood in front of a large screen (see image on lower left) on which the target, interception zone, starting point, and background of randomly distributed dots were presented. The background dots and interception zone were always visible. (B) The starting point was visible until shortly after the participant’s finger was placed on it, when the moving target appeared instead. About 357 ms later, the background started moving. The participants’ task was to lift their finger off the screen and tap on the target when it was within the interception zone. For clarity, in this schematic representation, the dots shift beyond the screen, but in reality, dots that moved out of the screen at one side were replaced by dots that appeared at the other side, so that there were always 500 randomly distributed dots on the screen. (C) The distribution of trials within the five blocks of each experiment. The first block of each experiment started with 10 unperturbed, practice trials.

In three experiments, we asked participants to tap on a moving target when it was within an interception zone (Fig 1). At a fixed moment during each trial, either 500 irrelevant background dots started moving together (which we will refer to as background motion) or the target jumped. Participants were not given any details regarding when or how often the background would move or the target would jump. Neither were they instructed as to how to deal with such perturbations. They were simply asked to tap on the targets. We examined how the way the participants’ movements were influenced by the perturbations changed during the course of the experiments. Since responding to background motion is counterproductive in terms of moving towards the position at which they intended to hit the target, we reasoned that participants might gradually learn to reduce the vigour of their adjustments in response to frequent background motion. Ideally, this reduction should be specific to background motion, so that the vigour of responses to the latest information about the target’s position remains appropriate for reaching the target. To evaluate how the response to new information about the target’s position is affected by repeated exposure to background motion, we also examined how movements were adjusted when the target jumped to a new position. Does the response to background motion gradually disappear? Does it do so without the responses to target jumps also disappearing?

In Experiment 1 we presented a long sequence of trials with background motion, assuming that this is optimal for learning to suppress responses. We tested responses to target jumps in additional trials before and after this sequence. In Experiment 2 we interleaved some trials with target jumps amongst many trials with background motion to see whether sometimes having to respond to a perturbation would prevent participants from suppressing responses to background motion. In Experiment 3 we expanded on this by having about equal numbers of trials with target jumps and background motion. We were mainly interested in whether participants would learn to suppress responses to irrelevant motion, but we also wanted to establish whether this came at the cost of not responding to target jumps.

### Participants

There were 36 different participants: 12 in each experiment. They all volunteered to take part despite being aware that they would receive no compensation for doing so. Four of the participants of Experiment 1, six of the participants of Experiment 2, and six of the participants of Experiment 3, were male. Two participants in each experiment were left-handed. All participants were between 18 and 30 years old, except for several older participants who took part in Experiment 1 (the oldest was 64 years old). Since we want to know whether people can learn to supress responding to background motion substantially, we only really need to test a few participants, but we considered it useful to get some impression of the variability in the extent to which people differ in their ability to suppress such responses, so we considered 12 participants per experiment to be a reasonable compromise between taking participants’ time and being confident about the findings. Before taking part in the experiments, participants were informed of the task and number of trials. If they still wanted to take part after that, they signed a consent form in accordance with our ethical approval (Ethische Commissie Bewegingswetenschappen; Protocol 2006−02). The data of Experiment 1 were collected between the 13^th^ and 23^rd^ of December 2022. Those of Experiments 2 and 3 were collected between the 21^st^ of March and the 2^nd^ of April 2024.

### Equipment

An Optotrak 3020 (Northern Digital, Waterloo, Ontario) was used to determine the position of an infrared marker that was attached to the nail of the index finger of the participant’s preferred hand (at 500 Hz). A second marker was briefly deactivated whenever a flash occurred on the screen, allowing us to synchronise the measured fingertip positions with target positions on the screen to within 2 ms. The images on the large screen (Techplex, 1.25 × 1 m; slanted 30° backwards) were back-projected at 120 Hz with a spatial resolution of 800 × 600 pixels using an In-Focus DepthQ Projector.

### Procedure

Participants were instructed to tap on the moving target when it was within the interception zone. There were no specific instructions about how to deal with the background motion. Each experiment consisted of 5 blocks of trials. Participants completed the five blocks within a single session, separated by short breaks. At the beginning of each block, participants placed their index finger on four sequentially presented points on the screen. This allowed us to use the measured position of the marker on the participant’s fingernail to relate where the participant considered their fingertip to be to positions on the screen. After this simple calibration, participants started each trial by placing their index finger on the starting point (Fig 1B). Between 600 and 1200 ms after the tip of the finger was placed within the boundaries of the starting point, the moving target appeared. It was a 2 cm diameter red disk that always appeared 40 cm above and 40 cm to the left of the starting point, moving rightward at 60 cm/s.

Once the target appeared, participants could start moving to intercept it. If they moved their finger too early, the target did not appear and they had to move their finger back to the starting point and wait for another 600–1200 ms for the target’s appearance. The 6 cm diameter circular region within which the participant was expected to try to hit the target was 40 cm above the starting point, so it took the target 667ms to reach the centre of the interception zone. Both the background and the interception zone were grey, but the interception zone was brighter. Apart from these items, there were also 500 blue dots, randomly distributed across the screen. Background motion consisted of these dots moving laterally together at 100 cm/s. Dots that moved off the screen were replaced by dots at the other side of the screen, so that there were always 500 dots randomly distributed across the whole screen. The dots were occluded by the target and starting point, but were visible as they moved across the interception zone. The dots started moving 357 ms after the target appeared. This moment is a compromise between moving the background as late as possible to increase response vigour [17], and ensuring that participants will usually tap more than 240 ms after background motion onset, because we anticipated wanting to present average responses for 240 ms. The chosen moment is 260 ms before the centre of the target entered the interception zone (from that moment, hitting the target was rewarded with a sound), and 310 ms before the centre of the target was at the centre of the interception zone.

In all three experiments, the first block started with 10 targets with no perturbation to get participants accustomed to the interception task (Fig 1C). They then completed 20 trials in which the target jumped 1 cm to the left, randomly interleaved with 20 trials in which the target jumped 1 cm to the right. These 40 trials were used to ascertain that participants compensated fully for changes in target position at the onset of the experiments. The rest of the first block and the other four blocks differed between the three experiments.

In Experiment 1, the first block continued with participants trying to tap on 100 targets in which the background moved to the left, randomly interleaved with 100 trials in which the background moved to the right. After this first block, there were three blocks of 200 trials with equal numbers of randomly interleaved trials with leftward and rightward background motion. In the final block there were 210 such trials, followed by 40 trials with equal numbers of randomly interleaved leftward and rightward target jumps. The total number of trials in Experiment 1 was therefore 1100.

In Experiments 2 and 3 all four kinds of trials were randomly interleaved after the first 50 trials. In Experiment 2, 5% of the perturbations were target jumps and 95% were background motion, with equal numbers of leftward and rightward perturbations for each. In Experiment 3, half the perturbations were target jumps, and the other half were background motion. In both cases, there were 150 such trials to complete the first block, and 200 in the following four blocks. The total number of trials in Experiments 2 and 3 was therefore 1000.

Participants received feedback about their performance. An acceleration threshold of 50 cm/s^2^ in the direction orthogonal to the screen and a maximal distance of 5 mm from the screen were used to determine when the finger tapped the screen. The target’s position at that moment was calculated, and if the position of the fingertip (as derived from that of the marker) at the moment of the tap was within the outline of the target, the target was considered to have been hit. If so, it remained visible at its position at the time of the tap for 500 ms. If the centre of the target was within the interception zone at that time, a sound indicated that the target had been hit successfully. If the target was hit while its centre was outside the interception zone, it stopped but no sound was presented. If the finger missed the target, the target moved away from the finger at 100 cm/s along a line connecting the positions of the target and fingertip at the moment of the tap.

### Analyses

**Fig 2:**
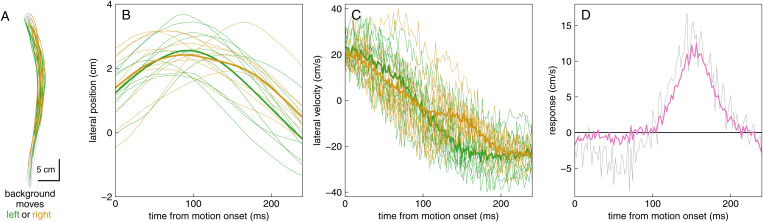
Determining the response to background motion. (A) Top view of the finger’s path on 20 trials of the third block for one of the participants of Experiment 1. The movements started at the starting point at the bottom, and ended when the finger tapped the screen at the top. The paths are coloured by the direction of background motion for 240 ms from when the background started moving. (B) The lateral position of the finger during these 240 ms (thin curves). The lateral position at each moment differs across trials, both because the paths differ and because the finger has moved different distances along the paths when the background starts moving. Nevertheless, the mean lateral positions for leftward and rightward background motion are initially quite similar (thick curves). (C) Considering the lateral velocity instead of the lateral position makes it easier to see that the background motion influences the finger’s movement after about 100 ms. (D) We use the difference between the mean lateral velocity in the trials in which the background moved to the right and in which the background moved to the left as our measure of the response to the background motion (grey curve). A positive response indicates that the finger moved in the direction of the background motion. The pink curve shows this participant’s response when all 200 trials of the block are considered, rather than only the 20 trials of panels A-C.

We were mainly interested in quantifying the extent to which the participants’ arms deviated in the direction of the background motion (i.e., the response). We did so on the basis of the lateral velocity of the finger during the first 240 ms from when the background started to move (which was about 357 ms after the target appeared), as explained in Fig 2. We determined the lateral velocity by dividing the displacement between consecutive measurements by the 2 ms time difference between them. Subsequently, we separately averaged the lateral velocity at each moment after leftward and rightward perturbations, and then determined the difference between these average velocities. This difference is the response (Fig 2D). We determined the response for each participant and block, and then averaged the response across participants to see how the response changed across blocks. A similar procedure was used to determine how the arm moved laterally in response to target jumps (considering the moment the target jumped and the direction in which it did so).

To get a more detailed impression of how the response amplitude decreases with practice, we also estimated how the vigour of the response to background motion decreased across sequential trials, irrespective of the block. To quantify the vigour of the response by a single number, we determined the mean lateral finger velocity from 100 to 200 ms after the background started to move for each trial. This time was chosen because we know from earlier experiments that the response will start a bit more than 100 ms after the background starts moving and will last at least 100 ms [16]. We determined separate weighted averages of this velocity across trials for each participant and direction of background motion using a moving Gaussian to determine the weights. We did so for steps of 1 trial and a standard deviation of 25 trials. The difference between the weighted averages for rightward and leftward background motion for each sequential trial is our estimate of the vigour of the response at that stage of the experiment.

Besides determining whether the vigour of the response to background motion decreases during the experiment, we also determined how the mean velocity profile of the fingertip as it moved up the screen changed during the experiment. For this, we only considered movement parallel to the screen, so learning to suppress lateral responses to background motion or to lift the finger higher or less high off the screen would not influence the outcome.

We also examined whether the percentage of targets that were hit increased during each experiment. If people are able to suppress the response to background motion, they might hit more targets. But an increase in the percentage of targets that are hit could also just be the result of learning how to best move the finger to perform the task. We therefore determined the percentage of targets that were hit within each block, both when the background moved and when the target jumped. If the percentage increases similarly across blocks for both kinds of perturbations, participants presumably just learn how to perform the task better. If the increase is more prominent for trials with background motion, the improvement is likely to be the result of responding less to background motion.

If participants learn to suppress responding to background motion, we need to make sure that they are specifically suppressing this response, rather than suppressing all responses during the movement. As already mentioned, we also examined the lateral response to target jumps. However, when there is a specified interception zone, target jumps are largely compensated for by changing the timing rather than the position of the tap [24]. Suppressing all responses, including changes in the timing of the tap, would probably lead to a decrease in the percentage of targets that are hit. But for a more sensitive measure, we also evaluated how well participants compensated for target jumps. We did so by determining the fraction of the change that they managed to compensate for on each trial. This fraction is 0 if there is no compensation: if they on average hit 1 cm ahead of the target centre for targets that jumped to the left, and 1 cm behind the target centre for targets that jumped to the right. The fraction is 1 if participants fully compensate for the target jump, on average hitting the target centre. For each participant and block, we determine the median value of this fraction. Note that this measure of compensation only captures the systematic error in responding to the jump, so full compensation does not necessarily mean that most targets were hit, nor that the target was on average hit at its centre. Beside also determining the fraction of targets that was hit, we therefore also determined where participants tapped, both with respect to the centre of the target and with respect to the centre of the interception zone. We determined the median lateral position for each participant in each block and then calculated the mean and 95% confidence interval across participants.

Responding vigorously to background motion cannot help performance, because it moves the arm away from its initial path. Responding vigorously to target jumps can be beneficial, because the timing or the path has to change in order to reach the target. If the vigour of the response is not selective for the kind of perturbation, we might expect to see it change after each trial: increasing after a target jump and decreasing after background motion. If so, we expect the vigour to remain higher in Experiment 3, where there were equal numbers of randomly interleaved trials with background motion and target jumps, than in the first two experiments. We also directly examined how the kind of perturbation on the previous trial influenced the response to background motion and to the target jumping. We split the data of Experiment 3 into two groups: trials following a trial with background motion and trials following a trial with a target jump. We averaged the vigour of the response to background motion and the extent of the compensation for target jumps separately for each participant for each of the two groups.

### Excluded trials

We did not differentiate between trials in which participants hit the target or missed it, or tapped the screen when the target was within or outside the interception zone. We only excluded trials if something went wrong technically (e.g., synchronization errors) or if the participant did not try to intercept the target, rotated their finger too far during the movement so that the marker was no longer visible, or tapped too soon. The target centre entered the interception zone (and was considered to be within the interception zone in terms of the feedback) after 617 ms, so on average only about 20 ms after the 240 ms time period within which we examined the velocity of the finger. Sometimes participants tapped the screen too soon: less than 240 ms after the perturbation. We excluded such trials from the plots of the responses (which extended for 240 ms), but we only excluded trials from the analyses of the vigour of the response if the tap occurred less than 200 ms after the perturbation (which did occasionally happen). With these criteria, we were able to use 98.4% of the trials of Experiment 1, 98.7% of those of Experiment 2, and 96.7% of those of Experiment 3 for the plots of the responses, and more of the data for the rest of our analyses.

### Statistical tests

At the end of the introduction, we posed two questions. The main question is whether the response to sudden, irrelevant background motion decreases with practice. An additional question, that is only relevant if the response to background motion does decrease, is whether the reduction in the response comes at the cost of a decrease in the response to relevant target jumps. We used one-tailed, paired t-tests to evaluate whether participants’ response magnitudes declined consistently from the first to the last block. We tested this separately for each of the three experiments. We also performed one-tailed paired t-tests to evaluate whether the vigour of the response and the extent of the compensation were larger after a trial with a target jump than after a trial with background motion in Experiment 3.

## Results

**Fig 3:**
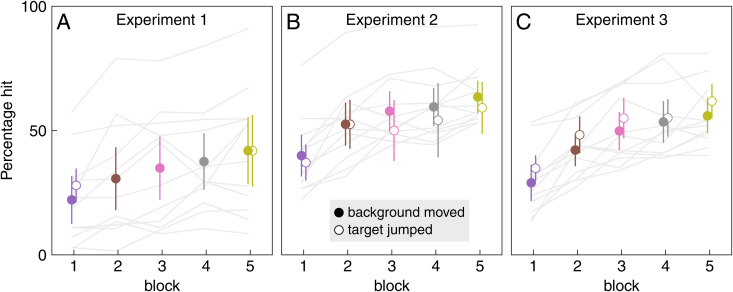
Percentage of trials that were hit. This is shown separately for trials in which the background moved (filled symbols) and ones in which the target jumped (open symbols), for each block of each experiment. There are no trials with target jumps in blocks 2 to 4 of Experiment 1 (see Fig 1C). Symbols and error bars show the mean and 95% confidence interval across participants. Grey lines show individual participants’ data.

In all three experiments, participants performed better as the experiments proceeded (Fig 3). There were more participants who performed poorly in Experiment 1 than in the other experiments, but we do not have an obvious explanation for this. In all experiments, both performance itself and the improvement in performance were similar when the background moved as when the target suddenly jumped, so we cannot attribute the improvement in performance to learning to suppress responding to background motion. Presumably, the improvement is the result of participants learning how to best move the finger to perform the task. Importantly, even in the last blocks of all experiments, participants still missed about half the targets, indicating that there was always room for improvement. One way to improve performance could have been by learning not to respond to the irrelevant background motion.

**Fig 4:**
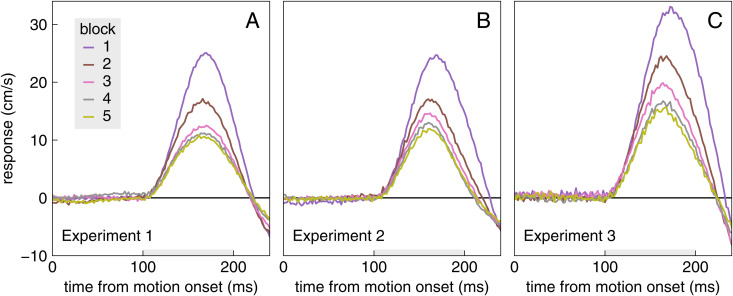
Average response to background motion in each block of each experiment. For details of how this is calculated see Fig 2. The grey bars indicate the time during which the response was averaged to quantify its vigour. Note that the target only reaches the centre of the interception zone 70 ms after the range that is shown (at t = 310 ms).

The average response to background motion decreased systematically across blocks (Fig 4). The response appears to be larger and to decrease more gradually in Experiment 3 than in Experiment 1, which makes sense because there are fewer trials with background motion from which to learn to supress the response to such motion in Experiment 3. To get a more detailed impression of how the response amplitude decreases with practice, we estimated how the vigour decreased across sequential trials, irrespective of the block.

**Fig 5:**
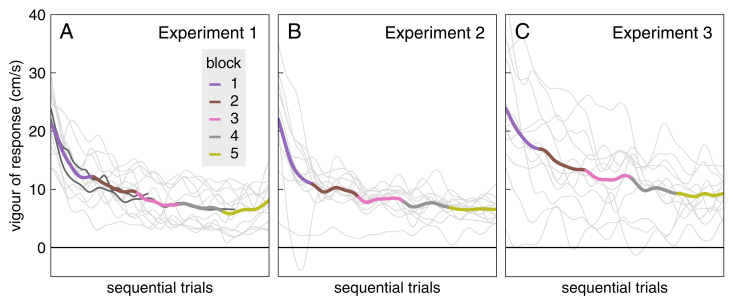
Average vigour of the response during sequential trials. Individual participants’ values are shown in grey. The average is coloured by the block of the trial at the position of the peak of the moving Gaussian that was used to calculate the smooth representation of the change in vigour. The two dark curves in A reproduce the thick curves of B and C, scaled horizontally to approximately represent the same numbers of sequential trials with background motion (so to 95% of the length of the curve for Experiment 2 and 50% for Experiment 3).

Almost all participants’ response vigour decreased during the experiment (Fig 5), indicating that they learnt to suppress the response to background motion to some extent. None of the participants managed to completely suppress the response (they did not end up with a response vigour of zero). There is quite a lot of variability across participants in the magnitude of the response. The variability is particularly prominent for Experiment 3, in accordance with the lower number of trials per point for Experiment 3. Having many interleaved trials in which the target jumped in Experiment 3 did not prevent participants from learning to suppress responses to background motion. The interleaved trials in which the target jumped hardly even influenced the rate at which the response magnitude decreased with practice, as can be seen by comparing the two dark curves with the thick coloured curve in Fig 5A. Thus, the response to background motion appears to mainly depend on how many trials with background motion one has encountered, irrespective of the number of interleaved trials with target jumps.

**Fig 6:**
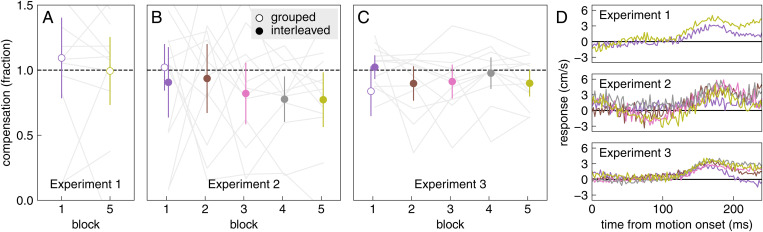
Median fraction of the target jump that is compensated for in each block of each experiment and lateral response to target jumps. The compensation (A-C) is determined separately for groups of successive trials with target jumps (at the beginning of the first block of each experiment and at the end of Experiment 1; open symbols), and for trials with target jumps that were interleaved amongst trials with background motion (solid symbols). The values are means and 95% confidence intervals of individual participants’ median fractions. Grey lines show individual participants’ data for the two groups of trials in Experiment 1 and for the interleaved trials of the other two experiments. The lateral response (D) is determined in the same manner as for the response to background motion onset (Fig 2), but considering the timing and direction of the jump rather than that of background motion. Colour coding as in Fig 5.

**Fig 7:**
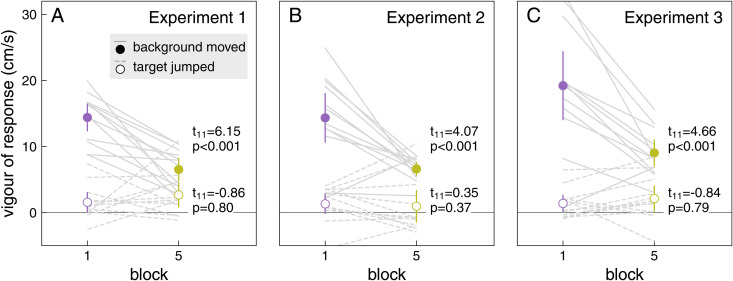
Change in response vigour between the first and last block. There was a significant reduction in the vigour of the response to background motion in all three experiments (solid lines and filled symbols; upper t-test in each panel). There was no systematic reduction in the vigour of responses to target jumps in any of the experiments (dashed lines and open symbols; lower t-test). Lines show individual participants’ data. Points show means with 95% confidence intervals across participants.

The decrease in response vigour with experience for background motion is not accompanied by less vigorous responses to target jumps. On average, in Experiment 1, participants fully compensated for target jumps after having been faced with over 1000 consecutive trials with background motion (Fig 6A, Block 5). If they had learnt to suppress all responses to perturbations, we would have expected them to respond substantially less vigorously to target jumps, in accordance with the decrease in vigour that can be seen in Figs 4A and 5A, and therefore to fail to compensate fully for the target jump. We see no evidence for systematic under-compensation in Experiment 1. There does appear to be a slight tendency to under-compensate for target jumps in Experiments 2 and 3 (Fig 6B and C), where some of the 95% confidence intervals (indicated by the error bars) do not include full compensation (indicated by the dashed line). Directly examining the lateral response to target jumps (Fig 6D) shows that the response to target jumps certainly does not decline across the blocks in the way that the response to background motion does. If anything, the response is weakest, rather than strongest, in the first session (purple curves). Consequently, while the response vigour to background motion declined significantly from the first to the last block, the response vigour to target jumps clearly did not decline (Fig 7).

**Fig 8:**
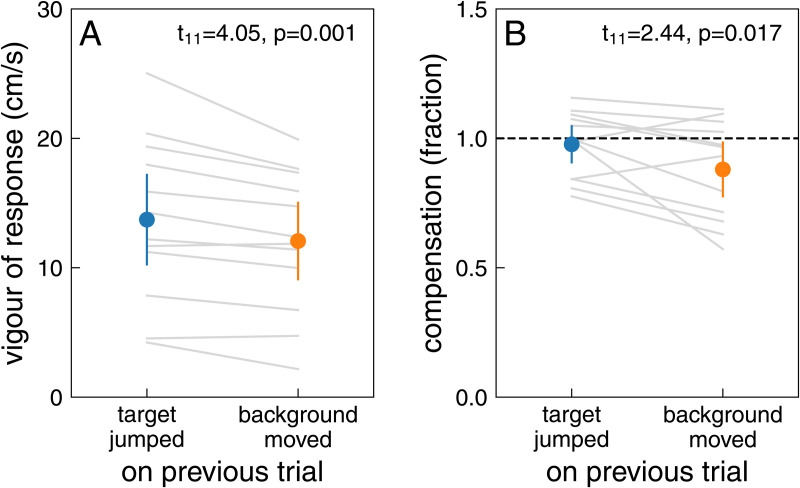
Influence of the previous trial in Experiment 3. How the previous target having jumped or the previous background having moved influenced the vigour of the response to background motion (A) and the median compensation for a target jump **(B)**. The symbols show means and 95% confidence intervals across individual participants’ values. Both the vigour of the response and the fraction compensated were significantly smaller when the background moved on the previous trial (one-tailed paired t-test). Grey lines show individual participants’ data.

A possible reason why compensation might be weaker in Experiment 2 than in Experiment 1 (compare Fig 6A and B), is that compensation might be slightly weaker on trials immediately following a trial with background motion. In Experiment 2, most trials with target jumps follow a trial with background motion, because most trials (95%) had background motion and the two kinds of perturbations were randomly interleaved. In block 5 of Experiment 1, the trials with target jumps are presented sequentially at the end, so only the first such trial follows a trial with background motion. To evaluate whether the kind of perturbation on the previous trial influences responses to the two kinds of perturbations, we split the trials of Experiment 3 by whether the target jumped or the background moved on the *previous* trial. The vigour of the response was lower (Fig 8A) and the compensation was less complete (Fig 8B) if the background moved on the previous trial. Thus, both the response to background motion and the response to target jumps depend on recent experience (Fig 8). But frequent exposure to target jumps hardly increases the response to background motion (as we saw by comparing curves in Fig 5A), so it is unlikely to be a single response vigour that is adjusted.

**Fig 9:**
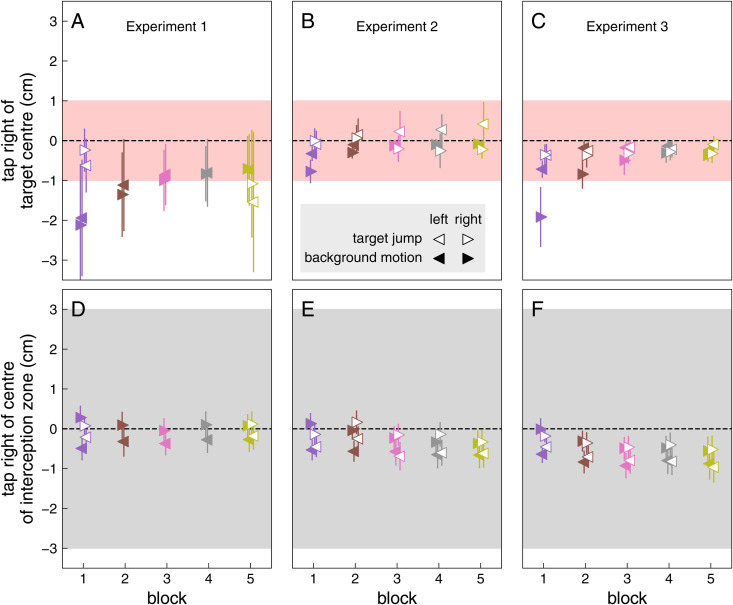
Median tapping errors with respect to the centres of the target and interception zone. The symbols show means and 95% confidence intervals across individual participants’ values. The pink (A, B, C) and grey (D, E, F) areas show the diameters of the target and interception zone, respectively.

**Fig 10:**
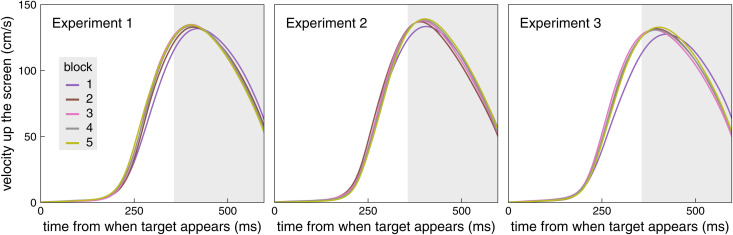
Velocity in the main direction of motion. The curves show the mean velocity across all trials of the indicated block, averaged across the participants’ average values. The main direction of motion is upwards, parallel to the screen and perpendicular to the perturbations. The velocity is shown from when the target appeared until the end of the period that we plot in Fig 4 (240 ms after the perturbation). The consistency between these curves is not strange, given the tight time constraints: participants were not allowed to start moving until the target appeared, and had to reach the target when it was within the interception zone, which was about 667 ms after it appeared.

Brief lateral motion of the finger in response to sudden background motion (Fig 4) might give rise to corrections that redirect the movement towards the centre of the target, and ensure that the target is hit within the interception zone. When the background moved, participants initially tended to tap to the left of the target centre (solid purple symbols in Fig 9A–C). This was especially so if the background moved to the right, in which case they also tended to tap slightly further to the right with respect to the interception zone (Fig 9D–F). Together, this means that participants must mainly initially tap to the left of the target because they tap too late. This is consistent with finding that although participants do not respond later in the first block than in subsequent blocks, they do initially move more slowly in the first block (purple curves in Fig 10). Participants do not correct fully for the response to background motion in terms of where they tap within the interception zone, but they do correct fully for the response to background motion in terms of where they tap with respect to the target (rightward pointing solid triangles are not systematically above leftward pointing ones in Fig 9A–C).

## Discussion

We wondered whether participants would learn not to respond to irrelevant background motion if they were repeatedly exposed to such motion. They learnt to respond less vigorously with repeated exposure, but the vigour of the response stopped decreasing well before there was no response at all (Figs 4 and 5). They learnt to respond less vigorously to background motion just as fast (in terms of the number of trials with background motion) when trials with background motion were interleaved with trials with target jumps, as when they were not (Fig 5A). The decrease in the vigour of the response to background motion was not accompanied by a decrease in the vigour of responses to target jumps (Fig 7). Thus, the vigour of responses to the two kinds of perturbations does not appear to be tightly coupled. However, there is some coupling, because the responses to both kinds of perturbations were slightly weaker immediately after a trial in which the background moved than immediately after a trial in which the target jumped (Fig 8). This might be why performance seems to be worse on trials in which the target jumped than on trials in which the background moved in Experiment 2 (Fig 3B), while it seems to be better in Experiment 3 (Fig 3C): the background usually moved on the previous trial in Experiment 2, so the average compensation is smaller (as seems to be the case according to Fig 6).

Even hundreds of consecutive trials with background motion were not enough for participants to learn not to respond to background motion at all. This was so despite the background motion being irrelevant to the task, and responding to such motion presumably being detrimental (in terms of requiring additional adjustments, so probably introducing more variability and costing more energy [25,26]). This confirms the suggestion that people are unable to completely ignore motion near their planned movement endpoint [21]. The reduction in the response is not the result of quickly correcting for the counterproductive response after it starts (as in [27]), because the difference between the response curves is visible as soon as the response starts (a bit more than 100 ms after the background starts moving; Fig 4). Thus, the whole response to background motion is suppressed. Maybe the response would have been suppressed more if the background motion had started later. In our experiments the background moved about 310 ms before the target reached the centre of the interception zone. So, there was usually enough time to largely counter the finger’s displacement as a result of the response to background motion before hitting the screen [11]. Consequently, participants only tapped slightly further to the right on the screen (i.e., relative to the centre of the interception zone) when the background pulled the finger to the right than when it pulled the finger to the left (Fig 9D–F). It is possible that if they had been unable to compensate for the finger following the background motion during the remaining time, for instance because the background had only started to move about 100 ms later, they would have learnt to suppress the response to background motion even further.

It is not yet clear why people cannot altogether avoid responding to motion of irrelevant items (near the planned movement endpoint [28]), but they also cannot avoid responding to irrelevant target displacements [13,14,29], despite quickly being able to select a different target [30,31]. People also cannot avoid following the displacement of obstacles on their path to a target when doing so is detrimental [32], despite being able to consider the instantaneous positions of obstacles when forced to adjust their movements [31,33,34]. Maybe these are all examples of responses to displacements being independent of responses to the new positions after the displacements [35]. Such independence would explain how responses to background motion can be supressed at all, without sacrificing the ability to adjust the movement when the target jumps, although the influence of the perturbation on the previous trial (Fig 8) shows that the responses to the two kinds of perturbations are not completely independent.

Another possible explanation is that the reduction in the vigour of responses to background motion that we find here results from learning to rely less on visual information from the image on the screen when updating the position towards which one is moving (that is ultimately defined with respect to oneself [36]). This would explain why it is impossible to completely avoid responding to background motion: it is impossible to rely exclusively on non-visual information to update the position towards which one is moving, because the judgment quickly becomes unreliable as one moves, or even only moves one’s eyes [37]. Although this explanation is highly speculative, it fits with other situations in which judged positions are influenced by shifts in items near movement endpoints, such as when items are shifted during saccades [38] or blank intervals [39,40]. One might particularly benefit from relying on non-visual information when intercepting a moving target, whereby the planned egocentric endpoint is not directly visible. However, this planned endpoint does not only need to be updated in response to one’s own movements, but must also constantly be reconsidered on the basis of new information about the changing position of the target [11]. The failure to completely ignore background motion might therefore arise from a compromise between learning to ignore the background, and drifting back towards the ‘natural’ situation of considering all the available visual information [37,41]. However, this explanation is inconsistent with the vigour of responses to target jumps not also decreasing across blocks (Fig 6D).

### Target jumps

The compensation for target jumps was incomplete in Experiments 2 and 3 (values lower than one in Fig 6), although there should have been enough time for full compensation [11]. There may not have been enough time to fully correct for the jump on some trials in which the participants tapped very early, but since we show the median compensation, that cannot explain this finding. The most likely cause is that having seen the background move on the previous trial reduced the gain of the compensation (Fig 8B). If one does not fully compensate for the jumps, one will tap to the left of targets that jump to the right and to the right of targets that jump to the left, as can be seen for the later blocks of Experiment 2 (positive values for leftward pointing open triangles and negative values for rightward pointing open triangles in Fig 9B). In general, the differences between the values for the two directions of target jumps (open symbols in Fig 9A–C) more or less correspond with the deviations from full compensation shown in Fig 6 (note that the values for the first block are split into grouped and interleaved trials in Fig 6B and C). But the two are not identical, as is most evident for block 5 of Experiment 1, where Fig 6A shows perfect compensation, while Fig 9A shows overcompensation for the target jumps (participants tapped further to the left when the target jumped to the left than when it jumped to the right). The two measures are not identical because the compensation is determined for each trial, considering the direction of the target jump on that trial. We then report the median compensation across all trials. The median position of the tap is determined separately for each direction of the target jump. Importantly, the overall interpretation is the same when considering both figures: there is a slight tendency for compensation to be incomplete for isolated interleaved target jumps (in accordance with Fig 8B).

In our analysis, we concentrated on the lateral component of the movement. However, the timing of the movement is also relevant, especially when the target jumps. Fig 9A–C reveals a bias to tap slightly to the left of the target centre, especially during the first block. There is also a tendency to move more slowly in the first block (Fig 10). A likely explanation for the bias to tap to the left of the target centre is therefore that participants more often tapped too late than too early. There is also indirect evidence that participants adjusted the timing of their movements. If participants had not responded to the target jumps, they would have hit 1 cm to the left of the target when the target jumped to the right, and 1 cm to the right of the target when the target jumped to the left. So the leftward pointing open triangles would be 2 cm higher (the height of the pink area) than the rightward pointing open triangles in Fig 9A–C. They are often slightly higher (compensation is often incomplete), but only slightly so, indicating that participants did compensate for the target jumps. One way in which they did so was by moving their fingers laterally in the direction in which the target jumped (Fig 6D). If they had fully compensated for the target jumps in this manner, they would have tapped 2 cm further to the right with respect to the interception zone when the target jumped to the right than when it jumped to the left. So the rightward pointing open triangles would be 2 cm higher than the leftward pointing open triangles in Fig 9D–F. The rightward pointing triangles are higher, but again only slightly so. The sum of the under-compensation and the lateral displacement never came close to 2 cm, so a substantial part of the compensation for the target jump must have been achieved by modifying the timing of the tap (as was to be expected when specifying an interception zone, because adjusting the timing is the only way one can hit the target centre when it reaches the centre of the interception zone; [24]). To fully compensate for the jumps by adjusting the timing of the tap, participants would have had to tap 17 ms earlier when the target jumped to the right, and 17 ms later if the target jumped to the left. Since about 4 mm was compensated for by moving the finger laterally (Fig 9D–F), and up to 6 mm was not compensated (Fig A-C), the timing of the tap was probably adjusted by between 9 and 14 ms.

## Conclusion

We set out to answer a simple question: can you learn not to respond to irrelevant motion while making fast arm movements? Strictly speaking, the answer appears to be ‘no’. None of our participants managed to completely stop responding to background motion. However, almost all the participants did clearly reduce the extent to which they responded to the irrelevant background motion. They did so without sacrificing their response to target jumps, so they did not simply suppress all responses. The more general answer to our question is therefore that participants can learn to specifically reduce the vigour of their responses to irrelevant motion, but that they cannot learn to stop responding to irrelevant motion altogether.
